# Syndecan-1 deficiency promotes tumor growth in a murine model of colitis-induced colon carcinoma

**DOI:** 10.1371/journal.pone.0174343

**Published:** 2017-03-28

**Authors:** Adi Binder Gallimidi, Gabriel Nussbaum, Esther Hermano, Barak Weizman, Amichay Meirovitz, Israel Vlodavsky, Martin Götte, Michael Elkin

**Affiliations:** 1 Sharett Oncology Institute, Hadassah-Hebrew University Medical Center, Jerusalem, Israel; 2 Institute of Dental Sciences, Hebrew University-Hadassah Faculty of Dental Medicine, Jerusalem, Israel; 3 Cancer and Vascular Biology Research Center, The Rappaport Faculty of Medicine, Technion, Haifa, Israel; 4 Department of Gynecology and Obstetrics, Muenster University, Medical Center, Muenster Germany; Duke University, UNITED STATES

## Abstract

Syndecan-1 (Sdc1) is an important member of the cell surface heparan sulfate proteoglycan family, highly expressed by epithelial cells in adult organisms. Sdc1 is involved in the regulation of cell migration, cell-cell and cell-matrix interactions, growth-factor, chemokine and integrin activity, and implicated in inflammatory responses and tumorigenesis. Gastrointestinal tract represents an important anatomic site where loss of Sdc1 expression was reported both in inflammation and malignancy. However, the biological significance of Sdc1 in chronic colitis-associated tumorigenesis has not been elucidated. To the best of our knowledge, this study is the first to test the effects of Sdc1 loss on colorectal tumor development in inflammation-driven colon tumorigenesis. Utilizing a mouse model of colitis-related colon carcinoma induced by the carcinogen azoxymethane (AOM), followed by the inflammatory agent dextran sodium sulfate (DSS), we found that Sdc1 deficiency results in increased susceptibility to colitis-associated tumorigenesis. Importantly, colitis-associated tumors developed in Sdc1-defficient mice were characterized by increased local production of IL-6, activation of STAT3, as well as induction of several STAT3 target genes that act as important effectors of colonic tumorigenesis. Altogether, our results highlight a previously unknown effect of Sdc1 loss in progression of inflammation-associated cancer and suggest that decreased levels of Sdc1 may serve as an indicator of colon carcinoma progression in the setting of chronic inflammation.

## Introduction

Syndecans comprise a major family of cell surface heparan sulfate proteoglycans (HSPGs) [1,2,3]. Among the four syndecans known in mammals, syndecan-1 (Sdc1) is the major HSPG found on epithelial cells, where its estimated expression reaches 10^6^ copies per cell [4]. Due to its high expression levels and ability to bind cytosolic, transmembrane, and extracellular matrix (ECM) proteins, Sdc1 plays important roles in numerous biological phenomena, including developmental processes, cytoskeleton organization, cell-cell and cell-ECM interactions [1,5,6,7,8]. Sdc1 mediates these processes, among other mechanisms, through binding of its heparan sulfate chains to ECM molecules and other effectors, including growth factors, cytokines, proteinases, and proteinase inhibitors [7,8,9,10,11,12,13]. The biological importance of Sdc1 is reflected by profound changes in its expression under several pathological conditions, including inflammatory and malignant diseases [7,8,11,12,13,14]. The gastrointestinal tract represents an important anatomic site where changes in Sdc1 expression were reported both in inflammation and malignancy. In the normal intestinal tract HSPGs are extensively found in the colon mucosa [15,16,17,18] and Sdc1 represents the predominant epithelial HSPG [19,20]. Due to its specific interaction with other ECM components, Sdc1 contributes to the structural integrity of the intestine as well as to electrostatic and mechanical barrier involved in the regulation of vascular and ECM permeability [18,21]. Indeed, Sdc1 deficiency was shown to cause protein leakage and to make the intestine more susceptible to proinflammatory cytokines and pressure [19]. Importantly, reduced levels of Sdc1 were found in the colon of patients with chronic inflammatory bowel disease (IBD) [22,23,24] and in a murine model of IBD [20,25,26]. Of note, Sdc1 deficient (*Sdc1-KO*) mice exhibit an enhanced inflammatory phenotype in a model of chemically induced acute colitis as evidenced by enhanced leukocyte infiltration, increased levels of inflammatory cytokines and adhesion molecules, and increased mortality [27]; however the effect of Sdc1 deficiency on chronic colitis was not investigated until recently.

The most feared long-term complication of chronic colitis is colon carcinoma, as patients with chronic colon inflammation have a risk of colorectal cancer which is an order of magnitude higher than the normal population [28]. Several studies linked Sdc-1 to colon tumorigenesis [29,30,31], however, unlike its involvement in inflammation, the role of Sdc-1 in colorectal cancer remains less unequivocal and, in light of the recent reports [32], is most likely context-specific. In a number of reports decreased epithelial Sdc1 expression was associated with an advanced clinical stage of colon tumors or poor prognosis [29,30], while one study associated epithelial Sdc1 immunopositivity with increased tumor size in colorectal carcinoma [32].

Our research was undertaken to elucidate the biological significance of Sdc1 in chronic colitis-associated tumorigenesis. Given the link between chronic inflammation and colorectal cancer [28], along with the changes in Sdc1 expression that were reported in both inflammatory and malignant disorders, we hypothesized that Sdc1 null mice would show altered susceptibility to chronic colitis-associated cancer. Here, utilizing mouse model of colitis-related colon carcinoma induced by the carcinogen azoxymethane (AOM), followed by the inflammatory agent dextran sodium sulfate (DSS) [33], we found that Sdc1 deficiency results in increased susceptibility to colitis-associated cancer, which was characterized by higher local production of IL-6 and activation of STAT3, as well as induction of several STAT3 target genes that act as important effectors of colonic tumorigenesis. Altogether our results highlight a previously unknown effect of Sdc1 loss in progression of inflammation-associated cancer and suggest that decreased levels of Sdc1 may serve as an indicator of colon carcinoma progression in the setting of chronic inflammation.

## Materials and methods

### AOM/DSS carcinogenesis model

**Fig 1:**
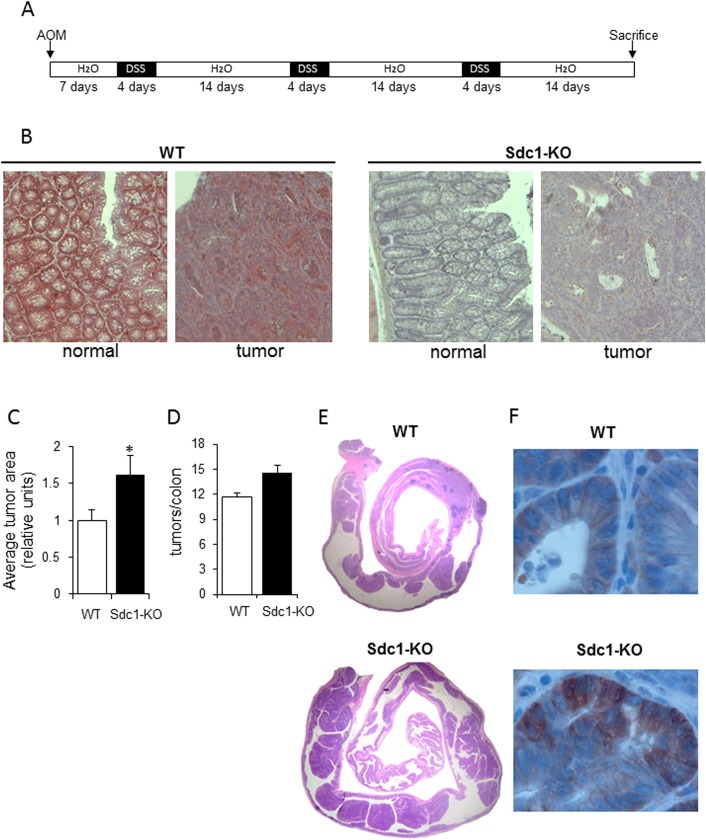
Syndecan-1 deficiency increases susceptibility to colitis-associated tumors in AOM/DSS-treated mice. **(A)** Schematic representation of the mouse model of AOM/DSS-induced colitis–associated carcinoma performed as described in Methods. **(B)** Representative immunostaining (reddish) for Sdc-1 in normal colonic tissue and tumor tissue samples derived from the colons of WT and Sdc1-KO mice. Original magnification X200. **(C)** Quantification of average tumor size, and **(D)** tumor number/colon in WT and Sdc1-KO mice on day 61 of AOM-DSS–induced colon cancer (n = 6). Error bars represent mean ± SE. *P < 0.05 by Student’s t test. **(E)** Representative histopathologic sections of colon adenocarcinomas from WT and Sdc1-KO mice. (F) Representative immunostaining for beta-catenin in tumor tissue samples derived from the colons of WT and Sdc1-KO mice. Original magnification X1000.

Male C57BL/6 mice were purchased from Harlan Laboratories (Jerusalem, Israel). Sdc1 knockout (Sdc-KO) mice [34] on a C57BL/6 background were bred at the animal facility of the Hadassah-Hebrew University Medical Center. All mice were kept under conventional pathogen-free conditions, and all experiments were performed with approval from the Hebrew University IACUC. Ten- to twelve-week old mice were injected (i.p.) with 10 mg/kg of AOM (Sigma-Aldrich). After a 7-day recovery period mice were administrated 1.5% DSS (Mr 36–40 kDa; MP Biomedicals) in the drinking water for 4 days, followed by 2-week consumption of regular water. This cycle was repeated 3 times (Fig 1A). Mice were sacrificed on experimental day 61 and their colons isolated and processed for histological examination and immunostaining, or snap-frozen for RNA isolation.

### DSS colitis model

Ten to twelve-week-old male mice were administrated 1.5% DSS in drinking water for 4 days, followed by 2-week consumption of regular water. This cycle was repeated 3 times. On indicated times mice were sacrificed, their colons isolated and processed for histological examination and immunostaining.

### Histopathology and immunostaining

Mouse colon was rolled up, fixed in 4% paraformaldehyde, and embedded in paraffin. Serial tissue sections (5 μm thick) were stained with H&E and visualized with a Zeiss axioscope microscope. The size of each neoplastic lesion was determined with Zeiss Image software (AxioVision) as previously described [35].

Histological scoring of inflammation was determined as described in [36] originated from [37]. Immunostaining of paraffin-embedded sections was performed as previously described [35]. Primary antibodies used were rat monoclonal anti-Sdc-1 (clone 281–2, Thermo Scientific), rabbit monoclonal anti-cyclin D1 (1:50, Thermo SCIENTIFIC), rabbit monoclonal anti-p-STAT3 (1:100, Cell Signaling), rat monoclonal anti-F4/80 (1:400, Serotec), mouse monoclonal anti-cMyc, rabbit polyclonal anti beta-catenin (1:400, Santa Cruz) and rabbit polyclonal anti-IL-6 (1:400, Abcam). Appropriate secondary antibodies (Nichirei) were then added and slides incubated at room temperature for 30 minutes. Color was developed using the DAB substrate kit (Thermo Scientific), or AEC (DAKO) for Sdc-1, followed by counterstaining with Mayer’s hematoxylin. Staining with the control IgG or without addition of primary antibody showed low or no background staining in all cases.

### Analysis of gene expression by qRT-PCR

Total RNA was isolated from snap-frozen tissue samples or cultured cells using TRIzol (Invitrogen), according to the manufacturer’s instructions, and quantified by spectrophotometry. After oligo (dT)- primed reverse transcription of 1 μg total RNA, the resulting single stranded cDNA was amplified using the primers listed below. Real-time quantitative PCR (qRT-PCR) analysis was performed with an automated rotor gene system RG-3000A (Corbett research, Sydney, Australia). The PCR reaction mix (20 μl) was composed of 10 μl QPCR sybr master mix (Finnzymes, Espoo, Finland), 5 μl of diluted cDNA (each sample in triplicate) and a final concentration of 0.3 μM of each primer. Hypoxanthine guanine phosphoribosyl transferase (HPRT) primers designed in the lab using Primer-BLAST software (NCBI) were used as an internal control. The following primers were utilized: mouse HPRT sense: 5′-GTC GTG ATT AGC GAT GAT GAA -3′, antisense: 5′-CTC CCA TCT CCT TCA TGA CAT C -3′, mouse IL-6 sense: 5′- AGC CAG AGT CCT TCA GAG AGA TAC-3′, antisense: 5′- GCC ACT CCT TCT GTG ACT CC-3′, mouse CCL-2 sense: 5'-GCATCCACGTGTTGGCTCA-3', antisense: 5'-AGCCTACTCATTGGGATCATCTTG-3', mouse cyclin D1 sense: 5'- AGCGGGAAGACCTCCTCTTC-3', antisense: 5'- ATCCGCCTCTGGCATTTTGG-3', mouse c-myc sense: 5'- TGAGCCCCTAGTGCTGCAT-3', antisense: 5'- AGCCCGACTCCGACCTCTT-3'.

## Results

To investigate the involvement of Sdc1 in colitis-related carcinoma, we applied the AOM-DSS mouse model of colitis-associated cancer [33]. Repeated oral administration of DSS-supplemented water to mice is a reliable model of chronic bowel inflammation (particularly, ulcerative colitis), recapitulating characteristic changes in the colon observed in human disease [38]. Single administration of DSS causes an acute inflammation in the colon [39], and we and others have previously shown that expression of Sdc1 is decreased in the DSS model and that Sdc1 deficiency results in an exacerbated acute inflammatory response [20,25,26,27]. When 2–3 cycles of DSS administration are applied, acute inflammation is followed by chronic colitis [39,40,41]. The mechanism by which DSS induces intestinal inflammation is likely the result of damage to the epithelial monolayer lining the large intestine allowing the dissemination of pro-inflammatory intestinal contents (e.g. bacteria and their products) into underlying tissue [38]. Importantly, chronic DSS-induced colitis subsequent to a single pretreatment with the carcinogen AOM results in development of colon tumors in ~100% of the treated mice [33]. Thus, to dissect the role of Sdc1 in colitis-related tumorigenesis, a single dose of AOM was injected to 10–12 week-old Sdc1-KO mice and their wild-type (wt) counterparts prior to 3 cycles of DSS treatment (as indicated in Fig 1A, and described in 'Methods'). Mice were sacrificed on day 61, their colon removed and examined for tumor formation. Colonic tumors (graded by histological examination as adenomas with high-grade dysplasia) were present in all colons (Fig 1B–1F). In agreement with previous reports [29,30], decreased Sdc1 levels were detected by immunostaining in the tumor, as compared to normal colonic tissue samples derived from wt mice (Fig 1B). As expected, no Sdc1 expression was detected in normal / tumor tissue samples derived from Sdc1-KO mice (Fig 1B). Importantly, a macroscopically apparent and statistically significant (p = 0.042) increase in tumor burden was easily detected in Sdc1-KO mice as compared to wt animals (Fig 1C and 1E). Notably, in the colon of Sdc1-KO mice larger pedunculated adenomas with villous architecture were abundantly present, while smaller, sessile adenomas were mostly found in the colon of wt mice. In addition, Sdc-1 deficiency resulted in an increase in tumor incidence (Fig 1D), although the differences between Sdc1-KO and wt groups did not reach statistical significance. Consistent with these findings, enhanced expression and shift of β-catenin from the cell membrane to a cytoplasmic/nuclear localization was detected by immunostaining in tumors derived from Sdc1-KO mice, as compared to wt mice (Fig 1F).

Numerous studies show that persistence and severity of inflammatory processes directly affect tumor growth in the colon [42]. Additionally, loss of Sdc1 expression is a characteristic feature of IBD both in experimental [20,25,26] and clinical [22,23,24] settings.

**Fig 2:**
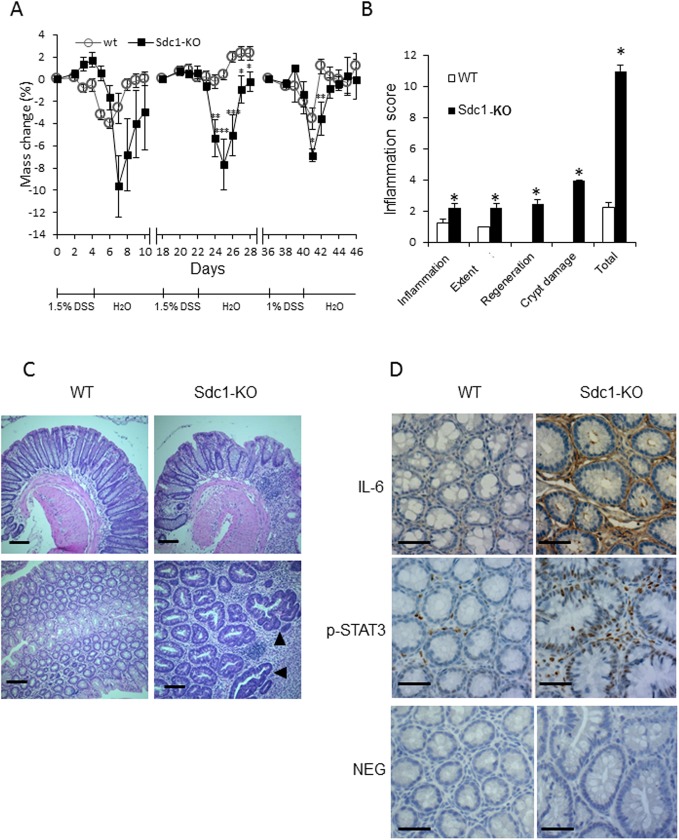
Syndecan-1 deficiency exacerbates colonic chronic inflammation in DSS treated mice. **(A)** WT and Sdc1-KO mice (n = 14 mice) were treated with three cycles of DSS as described in Methods and weight changes were measured. **(B)** Blinded histological scoring of inflammation in colonic mucosa of mice was performed as described in Methods. Error bars represent mean ± SE. *P < 0.05, **P < 0.01, **P < 0.001 by Student’s t test for mass change and Mann-Whitney U test for inflammation score.**(C)** Representative histopathologic sections of colon from WT and Sdc1-KO mice. Hyperplastic/dysplastic glands (lower panel—black arrowheads) were more frequent in Sdc1-KO than WT mice. Scale bar, 100 μm. **(D)** Representative immunoreactive staining (brown) for IL-6 (top panel) and phospho-STAT3 (middle panel) in colons of WT and Sdc1-KO mice. Scale bar, 100 μm. Lower panel: Representative negative controls (NEG) in which normal IgG was used in place of primary antibody.

Based on these notions, we next investigated whether the increased tumor size in Sdc1-KO mice is due to an exacerbated chronic inflammation in their colons. For this purpose, repetitive DSS cycles were administered alone, without AOM pretreatment. As shown in Fig 2, Sdc1 deficiency profoundly affected the chronic phase of DSS-induced colitis, as demonstrated by differences in the extent of weight loss and inflammation severity score between the groups (Fig 2A and 2B). In agreement, microscopic examination of H&E-stained colonic sections revealed increased mucosal infiltration by immunocytes in Sdc1-KO mice vs. wt mice (Fig 2C). This increase in infiltration was accompanied by morphologic changes typical for chronic inflammatory bowel disease (i.e., crypt architectural distortion) preserved in Sdc1-KO animals on day 33 (Fig 2C top panels) as well as occurrence of hyperplastic/dysplastic glands in colon of Sdc1-KO mice (Fig 2C bottom right panel, black arrowheads).

**Fig 3:**
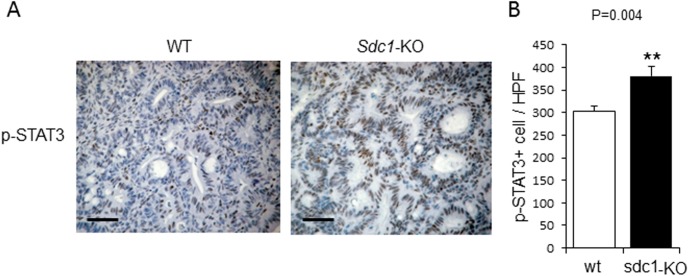
Increased activation of STAT3 in colonic tumors of Syndecan-1 KO mice compared to WT mice. **(A)** Representative immunoreactive staining (brown) for phospho-STAT3 in AOM-DSS induced colonic tumors (day 61) of WT and Sdc1-KO mice. Scale bar, 100 μm. **(B)** Quantification of average numbers of phospho-STAT3 positive cells per high power field (X400) in ≥ 12 fields of each slide from 4 mice of each group. Error bars represent mean ± SE. **P < 0.01 by Student’s t test.

**Fig 4:**
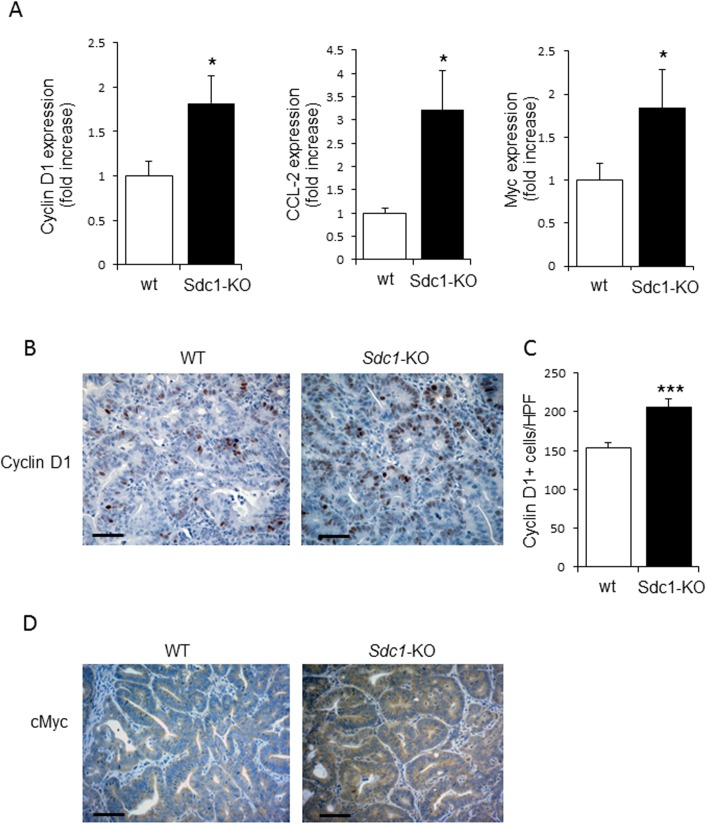
Elevated expression of pro-tumorigenic target genes of STAT3 in Syndecan-1 KO mice compared to WT. **(A)** Quantitative RT-PCR analysis revealed increased levels of Cyclin D1 (left), CCL-2 (middle) and Myc (right) in AOM-DSS induced colonic tumors derived from Sdc1-KO as compared to WT mice (n = 5). **(B)** Representative immunoreactive staining (brown) for Cyclin D1 in AOM-DSS induced colonic tumors (day 61) of WT and Sdc1-KO mice. Scale bar, 100 μm. **(C)** Quantification of average numbers of Cyclin D1 positive cells per high power field (X400) in ≥ 12 fields of each slide from 3 mice of each group. Error bars represent mean ± SE. *P < 0.05, ***P < 0.001 by Student’s t test. (D) Immunostaining for cMyc (brown) revealed increased levels of cMyc protein in AOM-DSS induced colonic tumors derived from Sdc1-KO vs. WT mice. Of note, cytoplasmic localization of cMyc was previously reported in several pathophysiological settings, including tumors of diverse origins (reviewed in [70]).

Moreover, as shown by immunostainting, an elevated expression of the proinflammatory cytokine IL-6 [which is tightly implicated in the pathogenesis of human and experimental chronic colitis [43,44,45]] was observed in DSS chronic colitis model in colons of Sdc1-KO as compared to wt mice (Fig 2D top panel). As shown in Fig 2D (top right), IL-6 positive cells were primarily found in the stromal compartment of the colon (e.g., lamina propria), in full agreement with previous reports identifying lamina propria-residing cells (i.e., macrophages) as principal producers of IL-6 in the setting of colitis-associated cancer [43,44,45]. Importantly, increased IL-6 expression levels were also detected in colonic tumors induced by AOM-DSS treatment in Sdc1-KO, as compared to wt mice (S1 Fig). As mentioned above, IL-6 is the major cancer-promoting cytokine in the setting of chronic colitis-associated carcinoma, which induces several pathways leading to tumor growth. One important mechanism of IL-6 procancerous action is activation of epithelial STAT3, a critical component of tumor-stimulating signaling in colon and other organs [44,45,46,47,48,49]. We therefore assessed the activation status of STAT3 in our system, applying immunostaining with anti-phosphoSTAT3 (pSTAT3) antibody. As shown in Fig 2D (bottom panels), a higher number of cells positive for nuclear-localized mucosal epithelial pSTAT3 was observed in the setting of DSS-induced chronic colitis in Sdc1-KO as compared to wt colon. Presence of pSTAT3 in cells located in lamina propria, in addition to epithelial cells (Fig 2 bottom right) is well documented in colitis-related cancer and reflects activated state of lamina propria-residing macrophages; yet, epithelial STAT3 activation was proven as the critical step in colitis-driven, IL-6 dependent tumorigenesis [44,47]. Importantly, increased activation of STAT3 was also demonstrated in AOM-DSS induced colonic tumors derived from Sdc1-KO, as compared to wt mice, as evidenced by a statistically significant increase (p = 0.004) in the number of cells positive for nuclear-localized pSTAT3 (Fig 3). In agreement with the well-established role of epithelial STAT3 in enhanced intestinal epithelial cell proliferation during colitis-associated tumorigenesis [44,47], elevated expression of the oncogene Cyclin D1 (a STAT3 target gene that plays a pivotal role in tumor growth [44] was detected in colonic tumors in Sdc1-KO vs. wt mice (Fig 4A left, 4B and 4C), providing a mechanistic explanation for the increased tumor burden (Fig 1B–1D). Accordingly, increased expression of additional STAT3 target genes critically involved in colon cancer progression, i.e., cytokine CCL-2 [36,50] and the oncogene Myc [51], was detected in Sdc1-KO tumors (Fig 4A, middle, right, 4D). Altogether these results suggest that Sdc1 loss promotes colonic tumor growth through augmented intestinal inflammation, activation of IL6-STAT3 signaling axis, and subsequent induction of Cyclin D1 and other pro-cancerous effectors.

## Discussion

Colorectal carcinoma represents a paradigm for the link between chronic inflammation and tumorigenesis [52,53]. In addition to extensively-documented changes in degree of immunocyte infiltration and levels of inflammatory cytokines in the course of cancer-promoting colonic inflammation, alterations in the expression of HSPG (in particular, decrease in Sdc1) were observed in both mouse and human studies [20,22,23,25]. While numerous reports have characterized the contribution of inflammatory cytokines (i.e., IL-6, TNFalpha) and their downstream transcription factors (NFkappaB, STAT3) in the development of inflammation-associated colon cancer (reviewed in [42,46,48,52,54,55,56]), limited data is available regarding the effect of altered levels of Sdc1 on the pathogenesis of colitis-associated tumors. To the best of our knowledge, this study is the first to test the direct effect of Sdc1 loss in colorectal tumor development in a model of inflammation-driven colon tumorigenesis.

For this investigation, we applied the AOM-DSS mouse model in Sdc1 KO and *wt* mice and demonstrated that Sdc1 deficiency resulted in increased susceptibility to colitis-associated tumorigenesis. As referred above, increased colonic inflammation is one potential mechanism that can contribute to the enhanced colon tumorigenesis in Sdc1 KO mice. Indeed, when a chronic DSS model was applied, Sdc1 KO mice featured an exaggerated and prolonged chronic inflammation that was characterized by distorted morphology of mucosa (closely resembling morphological changes typical for human IBD), increased IL-6 levels and activated STAT3 signaling, as compared to *wt* mice (Fig 2A–2D). Importantly, induction of IL-6 / STAT3 axis was also detected in colonic tumors derived from Sdc1 KO mice (S1 Fig, Figs 3 and 4). This signaling pathway is tightly implicated in colon carcinoma promotion, acting via induction of cell proliferation and inhibition of apoptosis (reviewed in [57]), in agreement with the observed increase in size of Sdc1 KO mice tumors (Fig 1C and 1E).

The majority of clinical studies have shown a positive association between decreased Sdc1 expression and colon tumorigenesis [29,30,31,58]. The role of Sdc1 in intestinal epithelial barrier integrity was described as well [19], and defects in the barrier function of intestinal epithelium (resulting in abnormal influx of gut microbial flora) are regarded as a hallmark in the pathogenesis of chronic inflammatory bowel disease [59]. In light of these observations and our present results, it is conceivable that in the setting of chronic inflammation-associated tumorigenesis loss of epithelial Sdc1 may facilitate access of luminal flora to the mucosal lymphoid tissue, thus creating chronic inflammatory conditions and contributing to tumor promotion. Interestingly, several Sdc1-KO mouse-based inflammation-related models, including our experimental system of DSS-induced chronic colitis, have generally shown exacerbation in inflammatory processes upon Sdc1 deficiency [27,60,61,62,63,64]. Mechanistically, it could be speculated that this phenotype is attributed to an augmented leukocyte recruitment in the absence of Sdc1, caused by increased interactions of leukocyte integrins with ICAM-1 [26,62]. Indeed, we have observed increased inflammatory cell recruitment in the DSS model (Fig 2). This increase may have caused increased IL-6 expression and secretion, which in turn leads to activation of STAT3 and a subsequent upregulation of downstream targets that promote tumorigenesis and tumor cell proliferation. Notably, increased leukocyte recruitment and increased expression of IL-6 and CCL2 in the absence of Sdc1 have been observed in other in vivo models of inflammation, including delayed-type hypersensitivity[63], glomerulonephritis [64], and autoimmune encephalomyelitis [65]. As Sdc1 can bind IL-6, and occasionally influence IL-6 signaling in a context-dependent manner, it has been discussed that the shed ectodomain may play an antagonistic role in this signaling pathway, leading to enhanced signaling in the absence of the proteoglycan (see [65] for discussion).

Surprisingly, a number of cancer models, utilizing Sdc1-KO mice and tumor types other than colon carcinoma, have shown a reduction in tumorigenesis. These effects were ascribed to reduced signaling of Wnt [66], HGF-MET [67,68], and VEGF [69] which are promoted by the intact Sdc-1 receptor. Of note, the experimental systems used in the above-mentioned studies [66] [67,68], [69] lack an inflammatory component. Thus, our in vivo results, mirroring the clinical observations, demonstrate a pro-cancerous net effect of Sdc1 loss in the setting of inflammation-related (colon) cancer. This observation is important for determining Sdc1 as an indicator for colon tumor progression, and may serve to explain some of the context-dependent effects of Sdc1 in different tumor entities. A limitation of our study is that later time points along the AOM-DSS induced colon tumorigenesis were not analyzed; such analyzes would potentially enable to test whether Sdc1 loss also affects tumor invasiveness, in addition to tumor growth, which is the focus of the present investigation.

Altogether, our findings suggest several pathways associated with Sdc1 loss that merit further exploration. Sdc1 loss results in increased inflammation and upregulation of IL-6 expression, which in turn leads to increased activation of STAT3, CCL2, Cyclin D1 and cMyc, thus driving a proliferative phenotype. Although the precise mechanisms underlying this association are not fully understood, loss of Sdc1 emerges as an indicator of augmented colon tumor progression in the setting of chronic inflammation. Finally, our results highlight the importance of flinging new strategies to interfere with Sdc1 loss for the treatment of disease.

## Supporting information

S1 Fig Increased IL-6 levels in colonic tumors of Syndecan-1 KO mice compared to WT. Quantitative RT-PCR analysis revealed a 9 fold increase in IL-6 mRNA levels in AOM-DSS induced colonic tumors derived from Sdc1-KO, as compared to WT mice (n = 5). Error bars represent mean ± SE. *P < 0.05 (Student’s t test).
(TIF)
