# Supplementary figures and images for: Syndecan-1 deficiency promotes tumor growth in a murine model of colitis-induced colon carcinoma
Source: PLoS One. 2017 Mar 28;12(3):e0174343. doi: 10.1371/journal.pone.0174343 (PMC5369774; doi:10.1371/journal.pone.0174343)

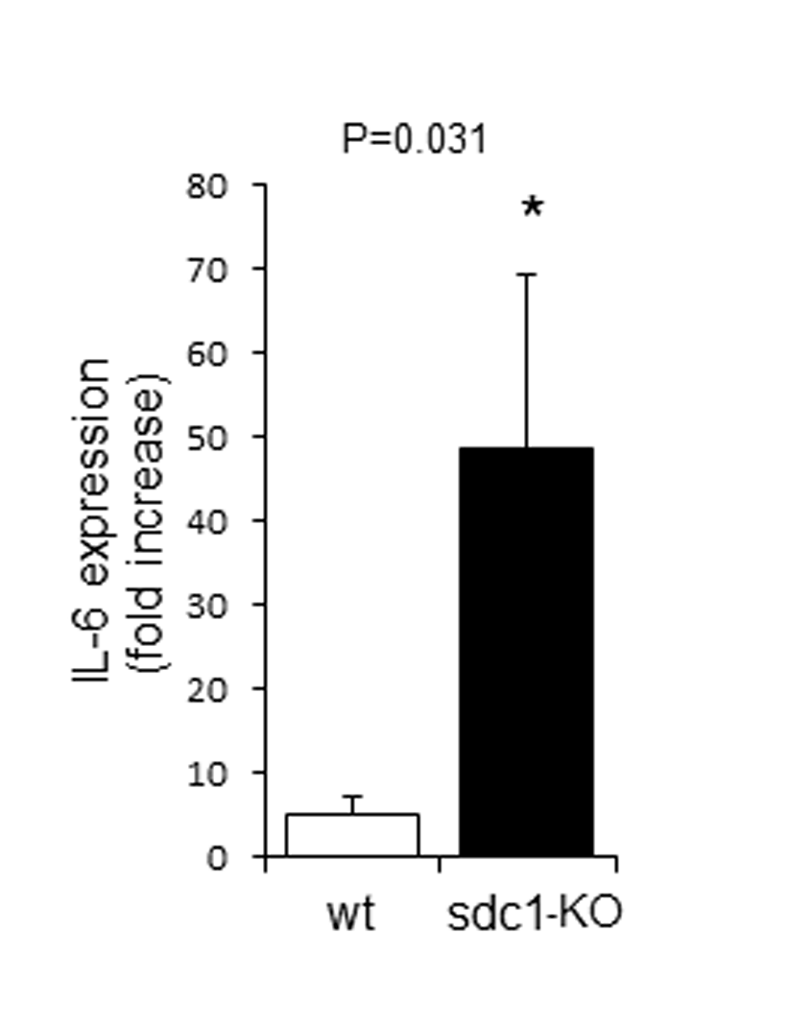

Supplement: S1 Fig — Quantitative RT-PCR analysis revealed a 9 fold increase in IL-6 mRNA levels in AOM-DSS induced colonic tumors derived from Sdc1-KO, as compared to WT mice (n = 5). Error bars represent mean ± SE. *P < 0.05 (Student’s t test). (TIF) [file pone.0174343.s001.tif]
